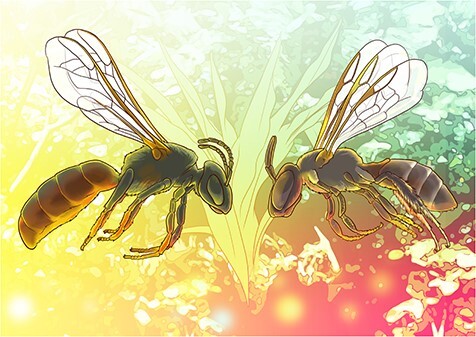# Battle of the bees: will native or invasive bees fare better with climate change?

**DOI:** 10.1093/conphys/coab080

**Published:** 2021-09-17

**Authors:** Lian Guo

**Affiliations:** Program in Organismic and Evolutionary Biology, University of Massachusetts Amherst, Amherst, Massachusetts 01003, USA

In Fiji, a battle ensues that mimics many ongoing invasions worldwide. The accidental introduction of two invasive bee species threatens the existence of the native bee, *Homalictus fijiensis*. Species that are highly successful at invading new environments tend to grow fast, reproduce rapidly and can tolerate broad temperature ranges. Thus, not only might the invasive bees outcompete *H. fijiensis* in obtaining food, but they may also be more tolerant of the new high-temperature patterns resulting from climate change.

Who will win the ‘Fiji Battle of the Bees’? More importantly, how will the outcome affect the pollination of local ecosystems?

While we could wait to see the results, there are faster ways to determine which bees will fare better under climate change. Scientists can challenge each species to extreme temperatures and humidity levels to see which species will maintain higher functioning and survival. Carmen da Silva and her colleagues did exactly this to predict how vulnerable each species will be to climate change. This team of scienstists expected that, because both invasive bees originate from environments with a broader range of temperatures and should have faster growth and reproductive rates, they would tolerate broader temperatures and use more energy than native *H. fijiensis* bees.

The researchers tested the thermal limits of each species. They first determined the high temperature that caused bees to stop moving. The team also tested the bees’ tolerance to dry conditions by exposing them to 5% relative humidity and determining when normal movements stopped. The researchers also measured the bees’ energy use (i.e. metabolic rate via CO_2_ production).

Unfortunately, both invasive bees were more tolerant of high temperatures and dry conditions than native *H. fijiensis* bees*.* Contrary to expectations, female *H. fijiensis* had higher resting metabolic rates than invasive *Braunsapis puangensis*. This means that *H. fijiensis* may invest more energy into reproduction than the invasive species. While higher reproductive investment could be a positive sign for the persistence of *H. fijiensis* populations, when food is scarce or competition with other species is significant, high energy demands require more time be spent to find food. Since *B. puangensis* and *H. fijiensis* forage on some of the same plant species, the combination of competition with higher energetic demands could lead *H. fijiensis* to be unable to maintain high reproductive rates and thus have lower population success.

The presence of invasive bees that have broad tolerance to temperature and humidity, in combination with direct climate impacts, make native *H. fijiensis* bees highly vulnerable to climate change. Conservation measures to protect *H. fijiensis* populations will be most effective if the combined impacts of climate change and invasive competitor bees are addressed. Native *H. fijiensis* bees forage on a greater diversity of plants than invasive *B. puangensis,* and so this means the potential loss in the pollination services of *H. fijiensis* may more negatively impact the local ecosystem than declines in invasive bee species.

If native *H. fijiensis* loses the battle of the bees in Fiji, yet another name will join the many extinct native species that have fallen victim to human-facilitated invasions and climate change.

**Illustrations**: Erin Walsh, ewalsh.sci@gmail.com